# Liquid Air

**Published:** 1898-04

**Authors:** 


### LIQUID AIR.

The article of our correspondent, W. H. T., on another page of this number, gives an interesting report of a lecture delivered by Professor George F. Barker, of the University of Pennsylvania, upon this new product obtained through the original method of Mr. Charles E. Tripler, of New York.

The report of this lecture created an intense curiosity in all circles, and great pressure was brought upon Professor Barker to have it repeated.

The writer had the pleasure of being present at this second lecture, March 14, and it is needless to say he held the interest of a closely packed audience from the beginning to the close.

The novelty of seeing air dipped up by a ladle from a can, not unlike, from outside appearance, to a milk-can, was rather astonishing to the auditors. The can resembled an ice-cream freezer in appearance. The inner can contained the fluid air, and between this and the outside can, heavy felt was packed.

The experiments which followed were all intended to show the intense degree of cold produced by the liquid air. Paraffine, when immersed in it, became in a few moments as white and as brittle as chalk. Rubber tubing was made as friable as glass. Tin would be affected similarly, but no effect was produced on copper. Beefsteak and an onion were frozen so thoroughly that they were readily crushed between the fingers. The familiar experiments with oxygen were repeated, showing the power of fluid air to support combustion. Mercury was frozen so hard that it became malleable under the hammer, and solid enough to drive a nail in a board.

While liquid air is not a new product, it has heretofore been produced only in small quantities, and at such great cost that it remained simply a scientific fact. Whether the certainty that it can be produced in unlimited quantities will make it of commercial value remains to be seen, but no doubt such will be the result.

That this production of liquefied air is one of the most interesting facts in modern science remains without dispute and it will no doubt soon be seen in all scientific educational circles, and like the Röntgen ray become common knowledge and, perhaps, like it, of equally practical value.

